# The longitudinal patterns of psychotropic drug prescriptions for subpopulations of community-dwelling older people with dementia: electronic health records based retrospective study

**DOI:** 10.1186/s12875-023-02021-9

**Published:** 2023-03-13

**Authors:** Jiamin Du, Sarah I. M. Janus, Michiel de Boer, Sytse U. Zuidema

**Affiliations:** 1 Department of General Practice and Elderly Care Medicine, University of Groningen, University Medical Centre Groningen, Groningen, the Netherlands; 2 Alzheimer Centre Groningen, Groningen, the Netherlands

**Keywords:** Dementia, Psychotropic drugs, General practice, Older people, Community dwelling

## Abstract

**Background:**

Studies focusing on patterns of psychotropic drug prescriptions (PDPs) for subpopulations of community-dwelling older people with dementia are lacking.

**Objective:**

The aim of this study was to identify the longitudinal patterns of PDPs in subpopulations.

**Methods:**

This retrospective study used electronic health records from general practitioners (GPs) in the Netherlands. People (*N* = 1278) firstly diagnosed with dementia between 2013 and 2015, aged 65 years or older, were selected and categorized into four subpopulations: community-dwelling (CD) group throughout follow-up, ultimately admitted to nursing homes (NH) group, ultimately died (DIE) group, and ultimately deregistered for unclear reasons (DeR) group. Generalised estimating equations were used to estimate the patterns of psychotropic drug prescriptions, after the diagnosis of dementia for a five-year follow-up, and 0–3 months before institutionalisation or death.

**Results:**

Over the five-year follow-up, antipsychotic prescriptions increased steadily in CD (OR = 1.07 [1.04–1.10]), NH (OR = 1.10 [1.04–1.15]), and DIE (OR = 1.05 [1.02–1.08]) groups. Similarly, prescriptions of antidepressants also showed upward trends in CD (OR = 1.04 [1.02–1.06]), NH (OR = 1.10 [1.02–1.18]), and DIE (OR = 1.04 [1.00–1.08]) groups. The other psychotropic drugs did not show clear changes over time in most of the subpopulations. In the three months before institutionalisation, antipsychotic prescriptions increased (OR = 2.12 [1.26–3.57]) in the NH group compared to prior periods. Likewise, before death, prescriptions of antipsychotics (OR = 1.74 [1.28–2.38]) and hypnotics and sedatives (OR = 2.11 [1.54–2.90]) increased in the DIE group, while anti-dementia drug prescriptions decreased (OR = 0.42 [0.26–0.69]).

**Conclusions:**

After community-dwelling older people are diagnosed with dementia, all subpopulations’ prescriptions of antipsychotics and antidepressants increase continuously during the follow-up. While we cannot judge whether these prescriptions are appropriate, GPs might consider a more reluctant use of psychotropic drugs and use alternative psychosocial interventions. Additionally, antipsychotic prescriptions rise considerably shortly before institutionalisation or death, which might reflect that older people experience more neuropsychiatric symptoms during this period.

**Supplementary Information:**

The online version contains supplementary material available at 10.1186/s12875-023-02021-9.

## Background

There are currently 290,000 people with dementia in the Netherlands, of whom about 79% live at home and are cared for by family caregivers [1]. Due to the aging-in-place reform, the Dutch population is encouraged to live at home for as long as possible [2]. Over the course of dementia, most people will experience neuropsychiatric symptoms (NPSs). NPSs are frequent and persistent in community-dwelling people with dementia [3]. A Dutch study showed that more than 90% of community-dwelling people with dementia had at least one NPS [4]. Taking care of older people with dementia and NPSs is challenging both physically and emotionally. NPSs not only increase caregivers’ burden, but also lead to institutionalisation [5, 6]. Adequate treatment of NPSs can help to reduce burden and postpone nursing home admissions. Psychosocial treatments are recommended as first-line therapy for NPSs [7–9]. Psychotropic drugs should only be used after non-pharmacological therapies failed [7–10].

Psychotropic drugs have many side effects, for example, antipsychotics may increase extrapyramidal symptoms, risk of cerebrovascular events and mortality, and antidepressants are associated with risks of falls and fractures [11, 12]. The use of psychotropic drugs in people with dementia has drawn researchers’ attention. However, most of the studies have focused on nursing home residents. The patterns of psychotropic drug prescriptions (PDPs) in nursing home residents may differ from community-dwelling people. Nursing home residents typically have more severe dementia than community-dwelling people and receive multidisciplinary care. Community-dwelling people have mild to severe dementia and are taken care of by their relatives, with possible support from home care helpers, case managers and general practitioners (GPs) [13]. Family caregivers may lack professional knowledge and coping strategies for NPSs. A qualitative study showed that family caregivers perceived psychotropic drugs to be generally safe and effective, and their use was associated with fewer barriers (cheaper and more resources) compared with the use of non-pharmacologic therapies [14]. These perceptions of family caregivers could influence GPs, leading to increased or earlier prescription of psychotropic drugs [15]. Insights into PDPs in community-dwelling older people with dementia can be a first step in optimizing future treatment for this group of patients.

There are some studies about the psychotropic drug use in community-dwelling people with dementia. A British cohort study reported that for community-dwelling older people who got the diagnosis of dementia between 1995 and 2011, 12.5% were prescribed antipsychotics, 22.1% antidepressants, 4.5% anxiolytics, and 9.8% hypnotics at the date of diagnosis [16]. A Finnish nationwide cohort study showed that among community-dwelling people with Alzheimer’s disease, 8.7% used antipsychotics, 19.2% antidepressants, and 21.2% benzodiazepines at the date of diagnosis [17]. Additionally, antipsychotic and antidepressant use increased steadily during a four-year follow-up [17]. The increases in the antipsychotic and antidepressant prescriptions were also reported in a longitudinal observational Dutch study, especially in the last follow-up year, the eighth year [18]. In this study, anxiolytic and hypnotic and sedative prescriptions were stable in the first seven years and increased in the eighth year [18]. These studies focused on community-dwelling people without considering subpopulations. However, community-dwelling people may have different PDP patterns, as some of them live in the community for a longer time, while others may move to nursing homes, or die during the follow-up. Studying patterns in subpopulations can help GPs understand what prescription patterns look like for people with different outcomes, especially nursing home admission or death. This could support them in reconsidering their prescription habits and in providing timely and sufficient support to possibly delay institutionalisation or help their patients to better cope with dementia at the end stage of life.

This study explored the patterns of PDPs in subpopulations of community-dwelling older people in two ways, during a five-year follow-up since the first diagnosis of dementia, and 0–3 months before institutionalisation or death.

## Methods

In this electronic health records (EHRs) based retrospective study, we used data from the Academisch Huisarts Ontwikkel Netwerk (AHON, Academic General Practitioners Development Network) database, which contains routinely collected health care data from about 50 GPs in the northern part of the Netherlands [19]. The data is pseudonymised. Diagnoses are encoded using the International Classification of Primary Care (ICPC-1) codes [20]. Medication prescriptions are encoded according to the Anatomical Therapeutic Chemical (ATC) classification [21]. In the Netherlands, GPs are gatekeepers in the health care system and have the authority to diagnose dementia and prescribe psychotropic drugs.

We identified 1350 people who got their first diagnostic code of dementia (P70) between 2013 and 2015. Their EHRs were extracted from one year before the diagnosis of dementia to five-year later, the end of this study (30^th^ November 2020), or the date of deregistration. Exclusion criteria were 1) aged younger than 65 when diagnosed with dementia, or 2) suffering from Down’s syndrome (A90.01). Additionally, we excluded people whose diagnosis date was later than the deregistration date.

### Demographic, administrative and clinical data

We extracted demographic data (date of birth in month and year, and gender), administrative data (registration dates at the GPs, deregistration dates and reasons), and clinical data (ICPC codes, ATC codes, and contact dates of every GP visit). Apart from dementia, the following psychological diagnoses and symptoms were extracted, in order to assess psychological comorbidities: feeling anxious (P01), feeling depressed (P03), feeling angry (P04), sleep disturbances (P06), memory disturbances (P20), delirium (P71.04), anxiety disorder (P74), and depressive disorder (P76).

The date of the first dementia diagnosis was considered the starting point of follow-up. The follow-up ended at the last date of the five-year follow-up for older people who continued living in the community, or the deregistration date for those who were admitted to nursing homes, died, or deregistered for unclear reasons during the follow-up. In case a patient had no visiting record for more than one year, we labelled this patient as deregistered for unknown reasons. The last contact date was used as deregistration date. We categorized the older people into four groups based on their registration status and deregistration reasons: community-dwelling (CD) throughout follow-up group, ultimately admitted to nursing homes (NH) group, ultimately died (DIE) group, and ultimately deregistered for unclear reasons (DeR) group which includes people who moved away for various reasons, or deregistered for unknown or unrecorded reasons.

### Primary outcome

The primary outcome was the presence (or absence) of a PDP every three months during follow-up. We distinguished five subgroups of psychotropic drugs: antipsychotics (N05A, excluding lithium and prochlorperazine), anxiolytics (N05B), hypnotics and sedatives (N05C), antidepressants (N06A), and anti-dementia drugs (N06D). We excluded lithium and prochlorperazine from antipsychotics because they are mainly used to treat bipolar disorder and severe vomiting, not dementia-related NPSs. In addition, prochlorperazine is not available on the Dutch market. All individual psychotropic drug prescriptions recorded in the database are shown in supplementary table 1. The Dutch College of GPs’ dementia guideline does not contain specific recommendations about the duration of PDP, but refers to the Dutch Association of Elderly Care Physicians (Verenso) guideline for problem behaviour in people with dementia [10, 22]. This guideline states that anxiolytics, hypnotics and sedatives should not be used for more than 4 weeks, and antipsychotics, antidepressants, and cholinesterase inhibitors should be tapered off gradually after 3 months [22]. Additionally, GPs in the Netherlands usually work with three-month prescription periods. Therefore, we chose three-month time intervals to longitudinally assess our outcome. The diagnosis date of dementia was used as the start date of follow-up. We compared the prescription date of the psychotropic drug with the diagnosis date of dementia to determine to which period this prescription belonged. This way a prescription belonged to a single period. We then transformed prescription data into dichotomous variables (presence or not) every three months for the five subgroups of PDPs separately. Prescriptions with a duration longer than three months were only counted in the first observed period, because we could not rule out the possibility of a record mistake. In practice, the duration of most prescriptions were less than 3 months. Additionally, if a prescription occurred multiple times in one period, it was only counted once.

### Statistical analyses

We used means and standard deviations, medians and interquartile ranges (IQRs) to describe continuous variables, and counts and percentages to describe categorical variables. We calculated the cumulative percentages of older people who were prescribed PDPs in each year and for the total five years.

Generalized estimating equations (GEE) were used to fit two repeated measures logistic regression models with exchangeable correlation structures, for the five subgroups of PDPs separately. Odds ratios (OR) and 95% confidence intervals (95% CI) were reported.

Model 1 aimed to estimate the patterns of PDPs among the subpopulations from the first diagnosis of dementia with a five-year follow-up. In this model, PDPs (assessed every three months) were the dependent variables. Variables indicating the time periods and the subpopulations (CD, NH, DIE, DeR) were included as independent variables. The model included both the main effects of these two variables and their interaction. The CD group was set as the reference group.

Model 2 aimed to estimate differences in PDPs 0–3 months before nursing home admission or death compared to prior periods within the subpopulation, and to people in the CD group. The PDPs were the dependent variables. A time-dependent event variable was included as independent variable. This variable was created based on deregistration reasons (nursing home admission, death, unclear) and deregistration time. The CD group was set as the reference and encoded value ‘0’ for all time periods because these older people lived in communities throughout the follow-up, i.e. no event occurred. For the other subpopulations, each subpopulation was encoded with two values, one value for 0–3 months before the event, and the other value for all prior periods. The details of the coding scheme are shown in supplementary table 2. To facilitate interpretation of the results, we calculated ORs within the NH and DIE group separately, comparing PDPs 0–3 months before the event with PDPs in prior periods (longer than three months before the event). We performed a sensitivity analysis using 0–6 months before events as the cut-off for time to the events. All statistical analyses were executed in SPSS version 25.

## Results

### Baseline characteristics

**Fig. 1:**
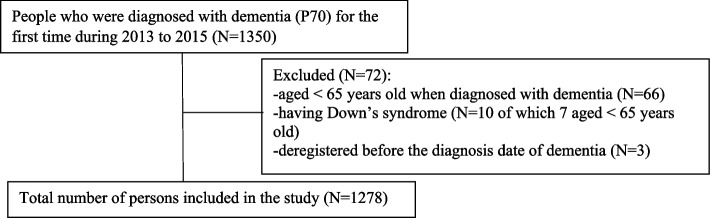
Flow chart for the inclusion of older people in the study sample

We included 1278 older people (Fig. 1). The numbers of and reasons for deregistrations during every three-month period throughout follow-up are shown in supplementary table 3. During the five-year follow-up, 391 people remained community-dwelling, 109 moved to nursing homes after a median of 29.7 months, 479 died after a median of 25.4 months, and 299 deregistered for unclear reasons after a median of 25.7 months (Table 1). The mean age at the time of the dementia diagnosis was 79.0 in the CD group, 81.9 in the NH group, and 84.2 in the DIE group. For the overall population, antipsychotics (5.48%) and antidepressants (6.65%) were the two most frequently prescribed psychotropic drugs in 0–3 months after the diagnosis of dementia. The cumulative percentages of older people who were prescribed PDPs are shown in supplementary table 4.

**Table 1:** Demographic characteristics, psychotropic drug prescriptions (0–3 months after the diagnosis of dementia), and psychological symptoms for the whole sample and for the subpopulations

	**Overall sample**	**CD group**	**NH group**	**DIE group**	**DeR group**
Sample size, N (%)	1278	391 (30.59)	109 (8.53)	479 (37.48)	299 (23.40)
Age at diagnose, Mean ± SD	81.96 ± 7.13	79.04 ± 6.48	81.90 ± 6.19	84.19 ± 7.17	82.22 ± 6.86
Gender, Female, N (%)	801 (62.68)	234 (59.85)	72 (66.06)	287 (59.92)	208 (69.57)
Follow-up months, Median (IQR)	38.92 (16.75–60.00)	60	29.73 (14.77–46.41)	25.36 (13.37–40.02)	25.69 (10.32–41.69)
Antipsychotics, N (%)	70 (5.48)	5 (1.28)	7 (6.42)	43 (8.98)	15 (5.02)
Anxiolytics, N (%)	50 (3.91)	16 (4.09)	4 (3.67)	17 (3.55)	13 (4.35)
Hypnotics and Sedatives, N (%)	49 (3.83)	17 (4.35)	3 (2.75)	25 (5.22)	4 (1.34)
Antidepressants, N (%)	85 (6.65)	30 (7.67)	4 (3.67)	28 (5.85)	23 (7.69)
Anti-dementia drugs, N (%)	38 (2.97)	13 (3.32)	3 (2.75)	12 (2.51)	10 (3.34)
Feeling anxious, N (%)	74 (5.79)	29 (7.42)	3 (2.75)	28 (5.85)	14 (4.68)
Feeling depressed, N (%)	69 (5.40)	29 (7.42)	2 (1.83)	25 (5.22)	13 (4.35)
Feeling angry, N (%)	62 (4.85)	20 (5.12)	4 (3.67)	22 (4.59)	16 (5.35)
Sleep disturbance, N (%)	152 (11.89)	66 (16.88)	7 (6.42)	54 (11.27)	25 (8.36)
Memory disturbance, N (%)	483 (37.79)	185 (47.31)	38 (34.86)	146 (30.48)	114 (38.13)
Delirium, N (%)	103 (8.06)	33 (8.44)	5 (4.59)	54 (11.27)	11 (3.68)
Anxiety disorder, N (%)	53 (4.15)	20 (5.12)	4 (3.67)	21 (4.38)	8 (2.68)
Depressive disorder, N (%)	110 (8.61)	49 (12.53)	3 (2.75)	36 (7.52)	22 (7.36)

*SD* standard deviation, *IQR* interquartile range

CD group: older people who continued living in the community during the 5-year follow-up

NH group: older people who moved ultimately to nursing homes during the 5-year follow-up

DIE group: older people who died ultimately during the 5-year follow-up

DeR group: older people who deregistered ultimately for unclear reasons during the 5-year follow-up

### The patterns of PDPs for the subpopulations during the five-year follow-up

**Fig. 2:**
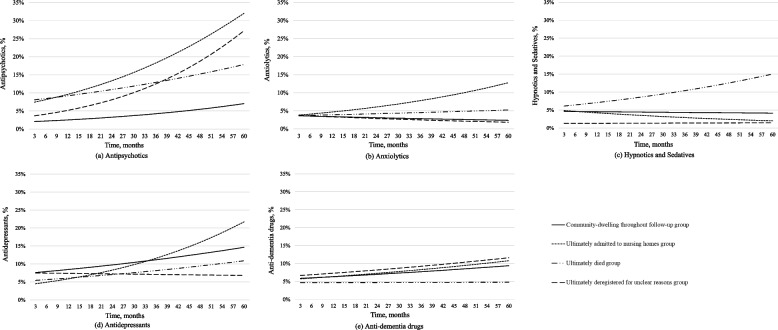
The estimated trends of the prescription of psychotropic drugs from diagnosis of dementia with a 5-year follow-up

We present the trends of PDPs for the four subpopulations during the five-year follow-up in Table 2 and as line charts in Fig. 2. The complete results of the GEE analysis (model 1) are shown in supplementary table 5. From the diagnosis of dementia onwards, prescriptions of antipsychotics increased every three months in all subpopulations: in the CD group with an OR of 1.07 (95%CI: 1.04–1.10), in the NH group with an OR of 1.10 (95%CI: 1.04–1.15), and in the DIE group with an OR of 1.05 (95%CI: 1.02–1.08). The antidepressant prescriptions increased over time as well, but at lower rates: in the CD group with an OR of 1.04 (95%CI: 1.02–1.06), in the NH group with an OR of 1.10 (95%CI: 1.02–1.18), and in the DIE group with an OR of 1.04 (95%CI: 1.00–1.08). There were no clear changes over time for most of the subpopulations with regard to the other classes of psychotropic drugs.

**Table 2:** The patterns of psychotropic drug prescriptions in subpopulations of community-dwelling older people during a five-year follow-up since the diagnosis of dementia, using three-month time intervals (*N* = 1278, Model 1)

	**Antipsychotics**	**Anxiolytics**	**Hypnotics and Sedatives**	**Antidepressants**	**Anti-dementia drugs**
	OR (95% CI)	OR (95% CI)	OR (95% CI)	OR (95% CI)	OR (95% CI)
**Time trends: Every 3 months**					
CD group	1.07^†^ (1.04, 1.10)	0.98 (0.95, 1.01)	0.99 (0.97, 1.02)	1.04 (1.02, 1.06)	1.03 (1.00, 1.05)
NH group	1.10 (1.04, 1.15)	1.07 (0.98, 1.16)	0.95 (0.92, 0.98)	1.10 (1.02, 1.18)	1.04 (0.97, 1.11)
DIE group	1.05 (1.02, 1.08)	1.02 (0.97, 1.07)	1.05 (1.02, 1.09)	1.04 (1.00, 1.08)	1.00 (0.96, 1.04)
DeR group	1.13 (1.09, 1.17)	0.96 (0.87, 1.06)	1.01 (0.90, 1.13)	0.99 (0.95, 1.04)	1.03 (1.00, 1.07)

*OR* odds ratio, *95% CI* 95% confidence interval

CD group: older people who continued living in the community during the 5-year follow-up

NH group: older people who moved ultimately to nursing homes during the 5-year follow-up

DIE group: older people who died ultimately during the 5-year follow-up

DeR group: older people who deregistered ultimately for unclear reasons during the 5-year follow-up

^†^ The odds of the antipsychotic prescriptions in the CD group increased, 1.07 times higher than the odds in the previous 3 months

### The patterns of PDPs 0–3 months before institutionalisation or death

In general, people in the NH and DIE groups had higher odds of antipsychotic prescriptions than in the CD group, which further increased 0–3 months before institutionalisation or death (Table 3). As an example for the interpretation of the ORs in Table 3, the odds of antipsychotic prescriptions in the NH group were 5.46 (95% CI: 3.00, 9.94) times higher in 0–3 months before the institutionalisation and 2.58 (95%CI: 1.43, 4.65) times higher in other previous periods compared with the odds in the CD group. This means that within the NH group, the odds of antipsychotic prescriptions in the period 0–3 months before institutionalisation was 2.12 (95%CI: 1.26–3.57) times higher than in previous periods (supplementary table 6). In 0–3 months before death, the odds of prescriptions of antipsychotics, hypnotics and sedatives increased with a factor 1.74 (95% CI: 1.28–2.38) and 2.11 (95% CI: 1.54–2.90) respectively compared to previous periods in the DIE group, whereas the odds of anti-dementia drug prescriptions decreased with a factor 0.42 (95% CI: 0.26–0.69). The sensitivity analyses results (supplementary table 7) showed that using a cut-off for the time period of 0–6 months before the events resulted in similar outcomes.

**Table 3:** The patterns of psychotropic drug prescriptions in 0–3 months before events and in other previous periods (> 3 months before events), compared with those in the CD group (*N* = 1278, Model 2)

	**Antipsychotics**	**Anxiolytics**	**Hypnotics and Sedatives**	**Antidepressants**	**Anti-dementia drugs**
	OR (95%CI)	OR (95%CI)	OR (95%CI)	OR (95%CI)	OR (95%CI)
Intercept^†^	0.04 (0.03, 0.06)	0.03 (0.02, 0.05)	0.05 (0.03, 0.06)	0.12 (0.09, 0.16)	0.08 (0.06, 0.11)
Time dependent status					
CD group	1	1	1	1	1
Was not admitted to NH yet	2.58^‡^ (1.43, 4.65)	1.88 (0.83, 4.25)	0.91 (0.40, 2.09)	0.67 (0.35, 1.27)	0.95 (0.49, 1.86)
Did not die yet	2.30 (1.50, 3.54)	1.39 (0.78, 2.47)	1.54 (0.97, 2.44)	0.59 (0.40, 0.89)	0.69 (0.44, 1.07)
Was not deregistered yet	1.42 (0.88, 2.29)	1.07 (0.55, 2.09)	0.29 (0.14, 0.62)	0.67 (0.42, 1.06)	1.11 (0.70, 1.75)
0–3 months before NH admission	5.46 (3.00, 9.94)	2.24 (0.93, 5.41)	0.83 (0.29, 2.39)	0.57 (0.25, 1.28)	0.59 (0.23, 1.53)
0–3 months before death	4.02 (2.60, 6.21)	1.28 (0.67, 2.42)	3.25 (2.11, 5.02)	0.50 (0.31, 0.79)	0.29 (0.15, 0.57)
0–3 months before deregistration for unclear reasons	2.61 (1.55, 4.39)	1.13 (0.53, 2.43)	0.30 (0.10, 0.84)	0.63 (0.37, 1.05)	0.65 (0.36, 1.19)

*OR* odds ratio; *95% CI* 95% confidence interval

CD group: older people who continued living in the community during the 5-year follow-up

^†^ The reported values of the intercept are odds with 95% confidence intervals

^‡^ The odds of antipsychotic prescriptions in the NH group in periods more than 3 months before institutionalisation were 2.58 times higher than the odds in the CD group

## Discussion

This was the first study reporting on patterns of PDPs in subpopulations of community-dwelling older people from the diagnosis of dementia until five years later. Antipsychotic and antidepressant prescriptions increased in the CD, NH, and DIE groups during the follow-up. Before nursing home admission, only antipsychotic prescriptions increased compared to prior periods. At the end of life, prescriptions of antipsychotics and hypnotics and sedatives increased, and prescriptions of anti-dementia drugs decreased.

### Patterns of antipsychotics and antidepressants

Antipsychotics and antidepressants were the two most frequently prescribed psychotropic drugs after the diagnosis of dementia. Agitation, depression, and irritability have been reported as the most common NPSs in Dutch community-dwelling older people with dementia [4]. These symptoms can be indications for the prescription of antipsychotics and antidepressants [22]. A high frequency of prescriptions of these two types of drugs were also found in other studies [16, 18].

Both classes of PDPs showed a steady increase in CD, NH, and DIE groups over time. The increase might be due to the progression of dementia and more frequent and severe accompanying NPSs. Other studies also reported increases in antipsychotics and antidepressants during the trajectory of dementia [17, 18]. We found that compared with older people who remained community-dwelling throughout the follow-up, those who ultimately moved to nursing homes or died had more antipsychotic prescriptions, which further increased 0–3 months before institutionalisation or death. Previous studies have shown that antipsychotics are associated with an increased risk of institutionalisation and mortality [23–26]. The increase in antipsychotics might be a result of more severe psychotic or agitated behaviour, or due to delirium superimposed on dementia [27–29]. NPSs could directly increase the risk of institutionalisation or death, or indirectly by increasing caregiver burdens [5, 23, 30, 31]. Additionally, using antipsychotics might be a risk factor in itself [23–26, 32]. Conversely, the percentage of antidepressant prescriptions in NH, and DIE groups were lower than in the CD group. The prescription rate of antidepressants did not change much 0–3 months before events. We hypothesize that dementia was at an advanced stage prior to nursing home admission. It has been reported that during the further progression of dementia towards severe stages, the prevalence of depression might decrease, although depression levels are difficult to assess in this population [3, 33, 34]. It is difficult to further speculate on possible reasons for the stable prescription rate of antidepressants, because the AHON registry does not include information on cause of death.

### Patterns of anxiolytics, hypnotics and sedatives

The prescriptions of anxiolytics, hypnotics and sedatives was stable during the follow-up. Joling et al. reported similar trends. [18] However, hypnotics and sedatives prescriptions almost doubled before death. Since the prevalence of sleeping disturbances in the DIE group was lower than in the CD group, we hypothesize that the increased hypnotics and sedatives prescriptions were probably due to palliative care in the terminal phase of life instead of insomnia. Hypnotics and sedatives might not only be used to treat sleeping problems, but to relieve suffering and provide comfort as well [35].

### Patterns of anti-dementia drugs

As found in a previous study, all subpopulations showed a slight increase in anti-dementia drugs over time [18]. The increase could be explained by the progression of dementia and deterioration in cognitive function. However, a reduction in anti-dementia drugs was observed before nursing home admission and especially before death. Arguably, people in NH and DIE groups generally had more severe dementia or poorer health status than those who remained living in the community, especially in the period prior to the events. Some of them might not react to anti-dementia drugs and these prescriptions could have been therefore stopped during the follow-up. Additionally, the prescriptions of acetylcholinesterase inhibitors or memantine may be inappropriate at the end of life, as any possible effect on cognition is not a relevant treatment goal anymore, and therefore, less likely to be prescribed [36–38].

## Strengths and limitations

A strength of our study is that we reported on the patterns of PDPs for subpopulations of older people who were ultimately admitted to nursing homes, died, or continued living in the community separately. In addition, we used two ways to estimate the patterns of psychotropic drugs, namely the longitudinal patterns during the five-year follow-up, and differences between 0–3 months before events and prior event-free periods.

Using EHR data has limitations, such as under-recorded diagnoses of dementia and deregistration reasons. It is therefore difficult to interpret the results for the DeR group. Another limitation is the lack of matched control groups. Therefore, we could not determine whether the patterns of PDPs were related to dementia or advanced age. However, this was also not a goal of our descriptive study. Furthermore, the severity of dementia and NPSs could not be obtained from the EHRs. Both characteristics are associated with institutionalisation [25–27]. In addition, the sample size of some subpopulations was small. Thus, these results might not be robust. Moreover, no information about the indications for PDPs and use of non-pharmacological interventions was available. Therefore, we cannot infer anything about the (in)appropriateness of PDPs. Finally, we analysed prescriptions rather than dispensations. The prescriptions might differ from drugs that people with dementia actually use.

## Conclusions

After the diagnosis of dementia, antipsychotic and antidepressant prescriptions in subpopulations increased continuously during a five-year follow-up. Although we had no information about the appropriateness of PDPs, the continuous increase might alert GPs to reconsider their prescribing habits and pay more attention to psychosocial interventions. Furthermore, antipsychotic prescriptions increased substantially in the period preceding nursing home admission or death. The increase in antipsychotics might reflect that patient experience more NPSs in this period. Future studies should examine how psychosocial interventions can be used in community-dwelling older people with dementia to decrease the need for psychotropic drugs.

## Supplementary Information

**Additional file 1:**

## Data Availability

The data that support the findings of this study are available from the Academic General Practitioner Development Network (AHON), but restrictions apply to the availability of these data, which were used under license for the current study. This means that the data are not publicly available but are available from the AHON committee upon reasonable request and with permission of the AHON committee.

## References

[1] Alzheimer Netherlands. Factsheet figures and facts about dementia. Updated February, 2021. https://www.alzheimer-nederland.nl/factsheet-cijfers-en-feiten-over-dementie. Accessed 3 June 2022.

[2] Maarse JAMH, Jeurissen PPP (2016). The policy and politics of the 2015 long-term care reform in the Netherlands. Health Policy (New York).

[3] Borsje P, Wetzels RB, Lucassen PL, Pot AM, Koopmans RT (2015). The course of neuropsychiatric symptoms in community-dwelling patients with dementia: a systematic review. Int Psychogeriatrics.

[4] Borsje P, Lucassen PLBJ, Wetzels RB, Pot AM, Koopmans RTCM (2018). Neuropsychiatric symptoms and psychotropic drug use in patients with dementia in general practices. Fam Pract.

[5] Isik AT, Soysal P, Solmi M, Veronese N (2019). Bidirectional relationship between caregiver burden and neuropsychiatric symptoms in patients with Alzheimer’s disease: a narrative review. Int J Geriatr Psychiatry.

[6] Backhouse T, Camino J, Mioshi E (2018). What do we know about behavioral crises in dementia?. A Systematic Review J Alzheimers Dis.

[7] Azermai M, Petrovic M, Elseviers MM, Bourgeois J, Van Bortel LM, Vander Stichele RH (2012). Systematic appraisal of dementia guidelines for the management of behavioural and psychological symptoms. Ageing Res Rev.

[8] Livingston G, Huntley J, Sommerlad A (2020). Dementia prevention, intervention, and care: 2020 report of the Lancet Commission. Lancet.

[9] Dyer SM, Harrison SL, Laver K, Whitehead C, Crotty M (2018). An overview of systematic reviews of pharmacological and non-pharmacological interventions for the treatment of behavioral and psychological symptoms of dementia. Int psychogeriatrics.

[10] NHG working group. NHG-Standaard Dementie (M21). Updated April, 2020. https://richtlijnen.nhg.org/standaarden/dementie. Accessed 1 July 2022.

[11] Wei YJ, Simoni-Wastila L, Lucas JA, Brandt N (2017). Fall and fracture risk in nursing home residents with moderate-to-severe behavioral symptoms of Alzheimer’s Disease and related dementias initiating antidepressants or antipsychotics. J Gerontol A Biol Sci Med Sci.

[12] Livingston G, Sommerlad A, Orgeta V (2017). Dementia prevention, intervention, and care. Lancet.

[13] Smits CHM, Van Den Beld HK, Aartsen MJ, Schroots JJF (2014). Aging in the Netherlands: state of the art and science. Gerontologist.

[14] Kerns JW, Winter JD, Winter KM, Kerns CC, Etz RS (2018). Caregiver perspectives about using antipsychotics and other medications for symptoms of dementia. Gerontologist.

[15] Jennings AA, Foley T, Walsh KA, Coffey A, Browne JP, Bradley CP (2018). General practitioners’ knowledge, attitudes, and experiences of managing behavioural and psychological symptoms of dementia: a mixed-methods systematic review. Int J Geriatr Psychiatry.

[16] Martinez C, Jones RW, Rietbrock S (2013). Trends in the prevalence of antipsychotic drug use among patients with Alzheimer’s disease and other dementias including those treated with antidementia drugs in the community in the UK: a cohort study. BMJ Open.

[17] Orsel K, Taipale H, Tolppanen AM (2018). Psychotropic drugs use and psychotropic polypharmacy among persons with Alzheimer’s disease. Eur Neuropsychopharmacol.

[18] Joling KJ, ten Koppel M, van Hout HPJ (2021). Psychotropic drug prescription rates in primary care for people with dementia from recorded diagnosis onwards. Int J Geriatr Psychiatry.

[19] Factsheet AHON-Database. Updated April, 2021. https://huisartsgeneeskunde-umcg.nl/system/files/factsheet_ahon-database_information_for_researchers.pdf. Accessed 3 June 2022.

[20] Bentsen BG (1986). International classification of primary care. Scand J Prim Health Care.

[21] WHO Collaborating Centre for Drug Statistics Methodology, Guidelines for ATC classification and DDD assignment, 2022. Oslo, 2021. https://www.whocc.no/atc_ddd_index_and_guidelines/guidelines/. Accessed 31 May 2022.

[22] Zuidema SU, Smalbrugge M, Bil WME, Geelen R, Kok RM, Luijendijk HJ, van der Stelt I, van Strien AM, Vink MT, Vreeken HL. Multidisciplinary Guideline problem behaviour in dementia. Verenso, NIP. Utrecht 2018.

[23] Brodaty H, Connors MH, Xu J, Woodward M, Ames D (2014). Predictors of institutionalization in dementia: a three year longitudinal study. J Alzheimer’s Dis.

[24] Kheirbek RE, Fokar A, Little JT (2019). Association between antipsychotics and all-cause mortality among community-dwelling older adults. Journals Gerontol - Ser A Biol Sci Med Sci.

[25] Schwertner  E, Secnik  J, Garcia-Ptacek  S (2019). Antipsychotic treatment associated with increased mortality risk in patients with dementia. a registry-based observational cohort study. J Am Med Dir Assoc.

[26] Nerius M, Johnell K, Garcia-Ptacek S, Eriksdotter M, Haenisch B, Doblhammer G (2018). The Impact of antipsychotic drugs on long-term care, nursing home admission, and death in dementia patients. Journals Gerontol - Ser A Biol Sci Med Sci.

[27] Addesi D, Maio R, Smirne N (2018). Prevalence of delirium in a population of elderly outpatients with dementia: a retrospective study. J Alzheimer’s Dis.

[28] Tremolizzo L, Bargossi L, Storti B, Ferrarese C, Bellelli G, Appollonio I (2021). Delirium in your house: a survey during General Practitioner-programmed home visits. Aging Clin Exp Res.

[29] Morandi A, Bellelli G (2020). Delirium superimposed on dementia. Eur Geriatr Med.

[30] Porter CN, Miller MC, Lane M, Cornman C, Sarsour K, Kahle-Wrobleski K (2016). The influence of caregivers and behavioral and psychological symptoms on nursing home placement of persons with Alzheimer’s disease: a matched case–control study. SAGE Open Med.

[31] Dufournet M, Dauphinot V, Moutet C (2019). Impact of cognitive, functional, behavioral disorders, and caregiver burden on the risk of nursing home placement. J Am Med Dir Assoc.

[32] Maust DT, Kim HM, Seyfried LS (2015). Antipsychotics, other psychotropics, and the risk of death in patients with dementia: number needed to harm. JAMA Psychiat.

[33] Zuidema SU, De Jonghe JFM, Verhey FRJ, Koopmans RTCM (2009). Predictors of neuropsychiatric symptoms in nursing home patients: influence of gender and dementia severity. Int J Geriatr Psychiatry.

[34] Holtzer R, Scarmeas N, Wegesin DJ (2005). Depressive symptoms in Alzheimer’s disease: natural course and temporal relation to function and cognitive status. J Am Geriatr Soc.

[35] Van Der Steen JT, Radbruch L, Hertogh CM (2014). White paper defining optimal palliative care in older people with dementia: a Delphi study and recommendations from the European association for palliative care. Palliat Med.

[36] Holmes HM, Sachs GA, Shega JW, Hougham GW, Cox Hayley D, Dale W (2008). Integrating palliative medicine into the care of persons with advanced dementia: identifying appropriate medication use. J Am Geriatr Soc.

[37] O’Brien JT, Holmes C, Jones M (2017). Clinical practice with anti-dementia drugs: a revised (third) consensus statement from the British association for psychopharmacology. J Psychopharmacol.

[38] François M, Sicsic J, Pelletier-Fleury N (2020). Determinants of antidementia drug prescription in patients older than 65: a latent class analysis. Pharmacoepidemiol Drug Saf.

