# Supplementary material for: The longitudinal patterns of psychotropic drug prescriptions for subpopulations of community-dwelling older people with dementia: electronic health records based retrospective study
Source: BMC Prim Care. 2023 Mar 13;24:69. doi: 10.1186/s12875-023-02021-9 (PMC10009999; doi:10.1186/s12875-023-02021-9)
Supplement: Supplementary file 1 — Additional file 1: [file 12875_2023_2021_MOESM1_ESM.docx]

**Supplementary Materials**

**S-Table 1** Overview of individual psychotropic drug prescriptions in patients with dementia in the AHON database (alphabetical order of ATC codes)

| **Subgroups of Psychotropic Drugs** | **3rd level ATC Codes** | **Psychotropic Drugs** | **Full ATC Codes** |
| --- | --- | --- | --- |
| Antipsychotics | N05A | Levomepromazine | N05AA02 |
|  |  | Haloperidol | N05AD01 |
|  |  | Pipamperon | N05AD05 |
|  |  | Zuclopentixol | N05AF05 |
|  |  | Clozapine | N05AH02 |
|  |  | Olanzapine | N05AH03 |
|  |  | Quetiapine | N05AH04 |
|  |  | Sulpiride | N05AL01 |
|  |  | Risperidon | N05AX08 |
| Anxiolytics | N05B | Diazepam | N05BA01 |
|  |  | Oxazepam | N05BA04 |
|  |  | Lorazepam | N05BA06 |
|  |  | Clobazam | N05BA09 |
|  |  | Alprazolam | N05BA12 |
|  |  | Hydroxyzine | N05BB01 |
|  |  | Buspiron | N05BE01 |
| Hypnotics and Sedatives | N05C | Nitrazepam | N05CD02 |
|  |  | Flunitrazepam | N05CD03 |
|  |  | Lormetazepam | N05CD06 |
|  |  | Temazepam | N05CD07 |
|  |  | Midazolam | N05CD08 |
|  |  | Loprazolam | N05CD11 |
|  |  | Zopiclon | N05CF01 |
|  |  | Zolpidem | N05CF02 |
|  |  | Melatonine | N05CH01 |
|  |  | Brotizolam | N05CM09 |
| Antidepressants | N06A | Imipramine | N06AA02 |
|  |  | Clomipramine | N06AA04 |
|  |  | Amitriptyline | N06AA09 |
|  |  | Nortriptyline | N06AA10 |
|  |  | Doxepin | N06AA12 |
|  |  | Maprotiline | N06AA21 |
|  |  | Fluoxetine | N06AB03 |
|  |  | Citalopram | N06AB04 |
|  |  | Paroxetine | N06AB05 |
|  |  | Sertraline | N06AB06 |
|  |  | Excitalopram | N06AB10 |
|  |  | Trazodon | N06AX05 |
|  |  | Mirtazapine | N06AX11 |
|  |  | Bupropion | N06AX12 |
|  |  | Venlafaxine | N06AX16 |
|  |  | Duloxetine | N06AX21 |
| Anti-dementia drugs | N06D | Donepezil | N06DA02 |
|  |  | Rivastigmine | N06DA03 |
|  |  | Galantamine | N06DA04 |
|  |  | Memantine | N06DX01 |
|  |  | Ginkgo folium | N06DX02 |

**S-Table 2** Example of the codes for time-dependent event variable in 0-3 months before events in model 2

| Patient / Measurement | M1,  1-3 months | M2,  4-6 months | M3,  7-9 months | M4,  10-12 months | M5,  13-15 months | M6,  16-18 months | … | M20,  58-60 months |
| --- | --- | --- | --- | --- | --- | --- | --- | --- |
| P1 | NH1 (1) | NH1 (1) | NH2 (2) | - | - | - | - | - |
| P2 | CD (0) | CD (0) | CD (0) | CD (0) | CD (0) | CD (0) | CD (0) | CD (0) |
| P3 | DIE1 (3) | DIE1 (3) | DIE1 (3) | DIE1 (3) | DIE1 (3) | DIE2 (4) | - | - |
| P4 | DeR1 (5) | DeR1 (5) | DeR1 (5) | DeR2 (6) | - | - | - | - |

M1: measurement period 1, which was done in 1-3 months; P1: patient 1;

NH1: did not move to nursing homes yet; NH2: 0-3 months before nursing home admission;

CD: still lived in the community at the end of the 5-year follow-up;

DIE1: did not die yet; DIE2: 0-3 months before death;

DeR1: did not deregister yet; DeR2: 0-3 months before deregistration for unclear reasons.

**S-Table 3** The study sample size and the numbers of and reasons for deregistration in each period

| **Follow-up period** | **Sample size, N** | **Deregistration reasons, N** | | |
| --- | --- | --- | --- | --- |
|  |  | **Admitted to NH** | **Died** | **Unclear reasons** |
| 0-3 months | 1278 | 8 | 44 | 27 |
| 3-6 months | 1199 | 4 | 33 | 29 |
| 6-9 months | 1133 | 5 | 20 | 15 |
| 9-12 months | 1093 | 4 | 22 | 27 |
| 12-15 months | 1040 | 7 | 28 | 16 |
| 15-18 months | 989 | 8 | 30 | 13 |
| 18-21 months | 938 | 8 | 28 | 9 |
| 21-24 months | 893 | 3 | 28 | 15 |
| 24-27 months | 847 | 3 | 31 | 13 |
| 27-30 months | 800 | 7 | 21 | 14 |
| 30-33 months | 758 | 11 | 29 | 11 |
| 33-36 months | 707 | 3 | 21 | 10 |
| 36-39 months | 673 | 4 | 23 | 9 |
| 39-42 months | 637 | 3 | 24 | 19 |
| 42-45 months | 591 | 3 | 17 | 16 |
| 45-48 months | 555 | 8 | 15 | 9 |
| 48-51 months | 523 | 6 | 15 | 13 |
| 51-54 months | 489 | 8 | 18 | 14 |
| 54-57 months | 449 | 6 | 14 | 13 |
| 57-60 months | 416 | 0 | 18 | 7 |

NH: nursing home.

**S-Table 4** The number of persons with prescriptions of psychotropic drugs during the five-year follow-up since the diagnosis of dementia **(**N, **%)**

|  | | **1^st^ year** | **2^nd^ year** | **3^rd^ year** | **4^th^ year** | **5^th^ year** | **5-year cumulative use** |
| --- | --- | --- | --- | --- | --- | --- | --- |
| **Antipsychotics** | |  |  |  |  |  |  |
|  | Overall Sample | 127 (9.94) | 109 (10.48) | 105 (12.40) | 86 (12.78) | 62 (11.85) | 306 (23.94) |
|  | CD group | 15 (3.84) | 19 (4.86) | 27 (6.91) | 37 (9.46) | 37 (9.46) | 66 (16.88) |
|  | NH group | 12 (11.01) | 15 (17.05) | 13 (20.97) | 8 (21.05) | 6 (30.00) | 33 (30.28) |
|  | DIE group | 72 (15.03) | 60 (16.67) | 41 (16.67) | 23 (15.97) | 12 (18.46) | 145 (30.27) |
|  | DeR group | 28 (9.36) | 15 (7.46) | 24 (16.22) | 18 (18.00) | 7 (14.89) | 62 (20.74) |
| **Anxiolytics** | |  |  |  |  |  |  |
|  | Overall Sample | 79 (6.18) | 63 (6.06) | 51 (6.02) | 37 (5.50) | 28 (5.35) | 149 (11.66) |
|  | CD group | 23 (5.88) | 19 (4.86) | 19 (4.86) | 19 (4.86) | 17 (4.35) | 49 (12.53) |
|  | NH group | 7 (6.42) | 8 (9.09) | 5 (8.06) | 3 (7.89) | 5 (25.00) | 13 (11.93) |
|  | DIE group | 28 (5.85) | 21 (5.83) | 19 (7.72) | 12 (8.33) | 4 (6.15) | 53 (11.06) |
|  | DeR group | 21 (7.02) | 15 (7.46) | 8 (5.41) | 3 (3.00) | 2 (4.26) | 34 (11.37) |
| **Hypnotics and Sedatives** | |  |  |  |  |  |  |
|  | Overall Sample | 94 (7.36) | 88 (8.46) | 71 (8.38) | 54 (8.02) | 42 (8.03) | 193 (15.11) |
|  | CD group | 31 (7.93) | 32 (8.18) | 30 (7.67) | 24 (6.14) | 28 (7.16) | 66 (16.88) |
|  | NH group | 8 (7.43) | 6 (6.82) | 1 (1.61) | 1 (2.63) | 1 (5.00) | 10 (9.17) |
|  | DIE group | 47 (9.81) | 43 (11.94) | 36 (14.63) | 25 (17.36) | 13 (20.00) | 99 (20.67) |
|  | DeR group | 8 (2.68) | 7 (3.48) | 4 (2.70) | 4 (4.00) | 0 (0.00) | 18 (6.02) |
| **Antidepressants** | |  |  |  |  |  |  |
|  | Overall Sample | 134 (10.49) | 109 (10.48) | 100 (11.81) | 99 (14.71) | 79 (15.11) | 218 (17.06) |
|  | CD group | 52 (13.30) | 53 (13.55) | 54 (13.81) | 66 (16.88) | 66 (16.88) | 91 (23.27) |
|  | NH group | 7 (6.42) | 9 (10.23) | 10 (16.13) | 8 (21.05) | 2 (10.00) | 17 (15.60) |
|  | DIE group | 44 (9.19) | 33 (9.17) | 27 (10.98) | 16 (11.11) | 8 (12.31) | 71 (14.82) |
|  | DeR group | 31 (10.37) | 14 (6.97) | 9 (6.08) | 9 (9.00) | 3 (6.38) | 39 (13.04) |
| **Anti-dementia drugs** | |  |  |  |  |  |  |
|  | Overall Sample | 110 (8.61) | 111 (10.67) | 85 (10.04) | 64 (9.51) | 52 (9.94) | 188 (14.71) |
|  | CD group | 36 (9.21) | 40 (10.23) | 39 (9.97) | 41 (10.49) | 45 (11.51) | 76 (19.44) |
|  | NH group | 10 (9.17) | 11 (12.50) | 9 (14.52) | 4 (10.53) | 1 (5.00) | 16 (14.68) |
|  | DIE group | 33 (6.89) | 31 (8.61) | 20 (8.13) | 12 (8.33) | 1 (1.54) | 51 (10.65) |
|  | DeR group | 31 (10.38) | 29 (14.43) | 17 (11.49) | 7 (7.00) | 5 (10.64) | 45 (15.05) |

CD group: older people who continued living in the community during the 5-year follow-up;

NH group: older people who moved ultimately to nursing homes during the 5-year follow-up;

DIE group: older people who died ultimately during the 5-year follow-up;

DeR group: older people who deregistered ultimately for unclear reasons during the 5-year follow-up.

**S-Table 5** Exponentiated regression coefficients of model 1, used for the construction of table 2, outlining drug prescriptions in subpopulations of community-dwelling older people (N=1278).

|  | **Antipsychotics** | **Anxiolytics** | **Hypnotics and Sedatives** | **Antidepressants** | **Anti-dementia drugs** |
| --- | --- | --- | --- | --- | --- |
|  | OR (95%CI) | OR (95%CI) | OR (95%CI) | OR (95%CI) | OR (95%CI) |
| **Intercept**† | 0.02  (0.01, 0.04) | 0.04  (0.02, 0.06) | 0.05  (0.03, 0.08) | 0.08  (0.06, 0.11) | 0.06  (0.04, 0.09) |
| **Time,**  **every 3 months** | 1.07  (1.04, 1.10) | 0.98  (0.95, 1.01) | 0.99  (0.97, 1.02) | 1.04  (1.02, 1.06) | 1.03  (1.00, 1.05) |
| **Events** |  |  |  |  |  |
| CD group‡ | 1 | 1 | 1 | 1 | 1 |
| NH group | 3,72  (1.59, 8,73) | 0.97  (0.35, 2.68) | 1.10  (0.45, 2.26) | 0.54  (0.23, 1.26) | 0.97  (0.42, 2.22) |
| DIE group | 4.15  (2.18, 7.92) | 0.98  (0.48, 1.99) | 1.27  (0.71, 2.26) | 0.70  (0.41, 1.19) | 0.81  (0.47, 1.40) |
| DeR group | 1.71  (0.83, 3.50) | 1.04  (0.45, 2.43) | 0.26  (0.11, 0.64) | 1.03  (0.59, 1.80) | 1.14  (0.66, 1.98) |
| **Time*Events** |  |  |  |  |  |
| Time * CD group | 1 | 1 | 1 | 1 | 1 |
| Time * NH group | 1.03  (0.97, 1.09) | 1.10  (1.00, 1.20) | 0.96  (0.92, 1.00) | 1.06  (0.98, 1.14) | 1.01  (0.94, 1.08) |
| Time * DIE group | 0.98  (0.94, 1.02) | 1.04  (098, 1.11) | 1.06  (1.01, 1.11) | 1.00  (0.96, 1.04) | 0.97  (0.93, 1.02) |
| Time * DeR group | 1.05  (1.01, 1.10) | 0.99  (0.89, 1.09) | 1.01  (0.90, 1.14) | 0.96  (0.92, 1.00) | 1.01  (0.96, 1.05) |

*OR*: odds ratio; *95% CI*: 95% confidence interval;

CD group: older people who continued living in the community during the 5-year follow-up;

NH group: older people who moved ultimately to nursing homes during the 5-year follow-up;

DIE group: older people who died ultimately during the 5-year follow-up;

DeR group: older people who deregistered ultimately for unclear reasons during the 5-year follow-up.

†: The reported values of the intercept are odds with 95% confidence intervals.

‡: The CD group was set as reference group.

**S-Table 6** Converted odds ratios (C-ORs) for psychotropic drug prescriptions, comparing 0-3 months before events with other previous periods within each subpopulation (N=1278, Model 2)

|  | | **Antipsychotics** | **Anxiolytics** | **Hypnotics and Sedatives** | **Antidepressants** | **Anti-dementia drugs** |
| --- | --- | --- | --- | --- | --- | --- |
|  | | C-OR (95%CI) | C-OR (95%CI) | C-OR (95%CI) | C-OR (95%CI) | C-OR (95%CI) |
| Time dependent status | |  |  |  |  |  |
|  | NH group | 2.12  (1.26, 3.57) | 1.20  (0.60, 2.37) | 0.91  (0.37, 2.26) | 0.86  (0.43, 1.72) | 0.62  (0.30, 1.29) |
|  | DIE group | 1.74  (1.28, 2.38) | 0.92  (0.58, 1.47) | 2.11  (1.54, 2.90) | 0.84  (0.61, 1.16) | 0.42  (0.26, 0.69) |
|  | DeR group | 1.83  (1.23, 2.72) | 1.06  (0.53, 2.09) | 1.02  (0.32, 3.24) | 0.94  (0.66, 1.34) | 0.59  (0.39, 0.90) |

*95% CI*: 95% confidence interval;

NH group: older people who moved ultimately to nursing homes during the 5-year follow-up;

DIE group: older people who died ultimately during the 5-year follow-up;

DeR group: older people who deregistered ultimately for unclear reasons during the 5-year follow-up.

**S-Table 7** The patterns of psychotropic drug prescriptions in 0-6 months before events and in other previous periods (>6 months before events), compared with those in the CD group (N=1278, Model 2)

|  | | **Anti-psychotics** | **Anxiolytics** | **Hypnotics and Sedatives** | **Antidepressants** | **Anti-dementia drugs** |
| --- | --- | --- | --- | --- | --- | --- |
|  | | OR (95%CI) | OR (95%CI) | OR (95%CI) | OR (95%CI) | OR (95%CI) |
| Intercept † | | 0.04  (0.03, 0.06) | 0.03  (0.02, 0.05) | 0.05  (0.03, 0.06) | 0.12  (0.09, 0.16) | 0.08  (0.06, 0.11) |
| Time dependent status | |  |  |  |  |  |
|  | CD group | 1 | 1 | 1 | 1 | 1 |
|  | Was not admitted to NH yet | 2.03  (1.02, 4.04) | 1.84  (0.78, 4.35) | 0.98  (0.42, 2.29) | 0.68  (0.35, 1.32) | 0.94  (0.47, 1.88) |
|  | Did not die yet | 2.14  (1.38, 3.31) | 1.27  (0.70, 2.30) | 1.50  (0.94, 2.41) | 0.57  (0.37, 0.86) | 0.68  (0.43, 1.08) |
|  | Was not deregistered yet | 1.15  (0.69, 1.91) | 1.13  (0.57, 2.23) | 0.30  (0.14, 0.62) | 0.61  (0.38, 0.98) | 1.09  (0.69, 1.73) |
|  | Half year before NH admission | 5.75  (3.29, 10.03) | 2.20  (0.94, 5.18) | 0.74  (0.25, 2.18) | 0.58  (0.26, 1.26) | 0.80  (0.36, 1.77) |
|  | Half year before death | 3.64  (2.40, 5.52) | 1.56  (0.88, 2.76) | 2.60  (1.70, 3.97) | 0.59  (0.39, 0.89) | 0.45  (0.26, 0.76) |
|  | Half year before deregistration for unclear reasons | 2.72  (1.69, 4.39) | 0.98  (0.47, 2.03) | 0.28  (0.12, 0.65) | 0.73  (0.46, 1.16) | 0.88  (0.53, 1.47) |

*OR*: odds ratio; *95% CI*: 95% confidence interval;

CD group: older people who continued living in the community during the 5-year follow-up.

†: The reported values of the intercept are odds with 95% confidence intervals.
